# Supplementary material for: Acute Pain Service in Hungarian hospitals
Source: PLoS One. 2021 Sep 22;16(9):e0257585. doi: 10.1371/journal.pone.0257585 (PMC8457450; doi:10.1371/journal.pone.0257585)
Supplement: S1 File — (DOCX) [file pone.0257585.s003.docx]

*Q 1. Hol található a kórház?*

a, Észak- Magyarország

b, Közép-Magyarország

c, Észak- Alföld

d, Dél-Alföld

e, Dél-Dunántúl

f, Nyugat-Dunántúl

g, Közép-Dunántúl

h, Budapest

*Q 2. Kitöltő személy beosztása:*

a, osztályvezető főorvos

b, szakorvos (milyen szakorvos:……………………………………………)

c, rezidens orvos

d, osztályvezető ápoló

e, osztályos ápoló

f, aneszteziológiai szakasszisztens

g, egyéb: (kérem, írja le!)……………………………………………………..

*Q 3. Mely típusú kórházban dolgozik?*

a, városi kórház

b, megyei kórház

c, egyetemi klinika

d, egyéb: (kérem, írja le!)………………………………………………………

*Q 4. Az intézmény fenntartója:*

a, Állami Egészségügyi Ellátó Központ (ÁEEK) által fenntartott intézmény

b, Magán fenntartású intézmény

c, Egyházi fenntartású intézmény

d, Emberi Erőforrások Minisztériuma által fenntartott intézmény

e, Egyéb fenntartású intézmény (kérem, írja le!):………………………………

*Q 5. Melyik osztályon dolgozik?*

a, aneszteziológia- intenzív terápiás osztály

b, fájdalom ambulancia

c, sebészet osztály

d, traumatológia osztály

e, ortopédia osztály

*Q 6. Melyik osztály vagy szolgálat foglalkozik a kórházban a műtét utáni fájdalom kezeléssel? (az összes igaz választ jelölje be)*

a, aneszteziológia és Intenzívterápiás osztály

b, Acute Pain Service (Akut Fájdalomkezelő Szolgálat)

c, fájdalom ambulancia

d, a beteget operáló osztály

e, Egyéb: (kérem, írja le!)……………………………………………………….

**APS KÉRDÉSSOR**

*Q 7. Működik-e Acute Pain Service (APS) team jelenleg a kórházban?*

a, igen

b, nem

**Amennyiben nemmel válaszolt, kérem, ugorjon a Q 26. kérdésre!**

*Q 8. Mióta van APS az intézményben? (Kérem, húzza alá!)*

a, kevesebb, mint 5 éve

b, több mint 5 éve

*Q 9. Hogy írná le az APS működését az intézményben? (Kérem, húzza alá!)*

a, küzdő, igyekvő team

b, virágzó, fejlődő

c, stabilan működő

*Q 10. Ki dolgozik az APS-ben? (az összes igaz választ húzza alá)*

a, aneszteziológus

b, aneszteziológiai szakasszisztens

c, gyógyszerész

d, sebész

e, neurológus

f, osztályos ápoló

g, kiterjesztett hatáskörű ápoló

h, pain nurse

i, gyógytornász

j, pszichológus

k, egyéb: (kérem, írja le!) ……………………………………………..

*Q 11. Hány aneszteziológus vesz részt az APS-ben? (Kérem, húzza alá!)*

a, 1

b, 2

c, 3 vagy több

e, egyéb(kérem, írja le!):……………………………………………………….

*Q 12. Hány aneszteziológiai szakasszisztens/nővér vesz részt az APS-ben? (Kérem, húzza alá!)*

a, 0

b, 1

c, 2

d, 3 vagy több

e, egyéb (kérem, írja le!):……………………………………………………………………

*Q 13. Az APS-ben dolgozó munkatársak kizárólag a posztoperatív fájdalom kezelését végzik? (azaz más munkát nem végeznek emellett) (Kérem, húzza alá!)*

a, igen, teljes munkaidős aneszteziológus

b, nem, részmunkaidős aneszteziológus más munkája mellett látja el ezt a feladatot is

c, igen, teljes munkaidős asszisztens/ápoló

d, nem, részmunkaidős asszisztens/ápoló más munkája mellett látja el ezt a feladatot is

e, egyéb: (kérem, írja le!)………………………………………………………………………..

Q *14. Az APS-ben dolgozó szakembereknek van-e lehetősége konzultálni a kórházban kábítószer vagy alkoholfüggő betegeket kezelő pszichiáterrel? (Kérem, húzza alá!)*

a, igen

b, nem

*Q 15. A nem sebészeti beavatkozáson átesett betegeket is ellátja az APS a munkája részeként (pl.szülészeti, daganatos, reumatológiai jellegű fájdalmak)? (Kérem, húzza alá!)*

a, igen

b, nem

*Q 16. Az APS munkájának mekkora részét teszi ki átlagosan a nem posztoperatív fájdalom kezelése? (pl.szülészeti, daganatos, reumatológiai jellegű fájdalmak) (Kérem, húzza alá!)*

a, 0-20%

b, 20-40%

c, 40-60%

d, 60-80%

e, több mint 80%

*Q 17. Hogy finanszírozzák az APS működését? (Kérem, húzza alá!)*

a, nincs rá külön finanszírozás

b, az aneszteziológia osztály kap rá külön finanszírozási keretet

c, az aneszteziológiai osztály nem kap rá külön keretet, saját keretéből kell kigazdálkodnia

d, sebészeti osztály kap rá finanszírozást

e, egyéb: (kérem, írja le!) ……………………………………………..

f, nem tudom

*Q 18. Van- e írott, a kórház felső vezetése által jóváhagyott megállapodás az APS és a műtétes osztályok között a betegek posztoperatív fájdalomcsillapító kezelésére vonatkozóan? (Kérem, húzza alá!)*

a, igen

b, nem

c, nem tudom

Amennyiben igen, kérem írja le a főbb tartalmi elemeit!..........................................................................................................................................

*Q 19. Az APS kezeli-e a műtét utáni fájdalmat valamennyi sebészeti beavatkozáson átesett betegnél vagy csak egy részüknél? (Kérem, húzza alá!)*

a, valamennyinél kezeli

b, csak egy részüknél kezeli

Amennyiben csak a betegek egy részét kezeli az APS, kérem, jelölje be, mely betegcsoportokat (az összes igaz választ jelölje be!)

a, az EDA kanüllel, IV-PCA-val rendelkező betegeket

b, a nagy hasi, mellkasi műtéten átesett betegeket

c, azon betegeket, akiknek a fájdalomcsillapító terápiája nem kielégítő az őt ápoló osztály jelzése alapján

d, egyéb: (kérem, írja le!)………………………………………………………………………..

*Q 20. Nyújt-e az APS szolgáltatást munkaidőn kívül?*

a, igen

b, nem

Amenyiben nem, kérem, adja, meg ki kezeli a nem kielégítően csillapított fájdalmat munkaidőn kívül?.............................................................................................................................

*Q 21. Nyújt-e az APS szolgáltatást hétvégén?*

a, igen

b, nem

Amennyiben nem, kérem, adja, meg ki kezeli a nem kielégítően csillapított fájdalmat hétvégén?...........................................................................................................

*Q 22. Használnak-e külön APS megfigyelőlapot az intézményben?*

a, igen

b, nem

Ha igen, kérem írja le a főbb tartalmi elemit!.................................................................................

*Q 23. Gyűjtenek-e adatokat az APS működéséről? (Kérem, húzza alá!)*

a, igen

b, nem

c, nem tudom

Amennyiben igen, milyen módon gyűjtik? (Kérem, húzza alá!)

a, papír alapon

b, elektronikusan (saját fejlesztésű vagy vásárolt program segítségével)

c, mindkettő

*Q 24. A keletkezett adatokat megosztják-e valakivel? (Kérem, húzza alá!)*

a, igen

b, nem

Ha megosztják az adatokat, az kivel történik? (az összes igaz választ jelölje be!)

a, kórház vezetése

b, munkatársak

c, műtétes osztályok képviselői

d, más kórházak

e, egyéb: (kérem, írja le!)………………………………………………..

*Q 25. A beteglátogatásokra vonatkozóan gyűjtenek-e adatot? (Kérem, húzza alá!)*

a, igen

b, nem

A mennyiben gyűjtenek, milyen fajtát? (az összes igaz választ jelölje be!)

a, beteg neme, életkora

b, műtét fajtája, típusa (elektív, sürgős)

c, beteg ASA besorolása

d, bemetszés helye

e, anesztézia típusa

f, beteg fájdalom pontszámai

g, anesztéziával, postoperativ fájdalomcsillapítással összefüggő nemkívánatos események száma, fajtája

h, a technika minősége

i, egyéb: (kérem, írja le!)……………………………………………..

*Q 26. Amennyiben nem működik APS az intézményben, érzi-e szükségét a kórházban APS kialakításnak? (Kérem, húzza alá!)*

a, igen

b, nem

*Q 27. Mi a legnagyobb akadálya az APS elindításnak? (az Önnek legfontosabb 4 igaz választ húzza alá!)*

a, nem elérhető gyógyszerek, eszközök

b, finanszírozás hiánya

c, kezdeményezés hiánya, motiváció hiánya

d, támogatás hiánya a menedzsment részéről

e, érdeklődés hiánya a kollégák részéről

f, a társszakmák együttműködésének elégtelensége

g, humán erőforrás hiánya

h, egyéb: (kérem, írja le!)…………………………………………………………………

*Q 28. A jövőben tenne-e aktívan a kórházban egy APS elindításáért? (Kérem, húzza alá!)*

a, igen

b, nem

c, nem válaszol

*Q 29. Véleménye szerint az aneszteziológusoknak kellene felelősséget vállalni a kórházakban az APS-ek működéséért? (Kérem, húzza alá!)*

a, igen

b, nem

Bármely válasz esetén, kérem, indokolja néhány mondatban válaszát!

………………………………………………………………………………………………….
