# Supplementary material for: Acute Pain Service in Hungarian hospitals
Source: PLoS One. 2021 Sep 22;16(9):e0257585. doi: 10.1371/journal.pone.0257585 (PMC8457450; doi:10.1371/journal.pone.0257585)
Supplement: S2 File — (DOCX) [file pone.0257585.s004.docx]

Questionnaire

1. **Where can the hospital be found?**

a, North Hungary

b, Central Hungary

c, Northern Great Plain

d, Southern Great Plain

e, Southern Transdanubia

f, Western Transdanubia

g, Central Transdanubia

h, Budapest

1. **Position of respondent professionals**

a, Head of department

b, Specialist (what type………………..)

c, Doctor in residency training

d, Headnurse

e, Ward nurse

f, Anesthesiology assistant

g, Other (please specify………………..)

1. **What type of hospital do you work at?**

a, City hospital

b, County hospital

c, University clinic

d, Other (please specify…………………….)

1. **Ownership**

a, Governmental hospital

b, Private hospital

c, Church-owned hospital

d, Ministry of Human Capacities hospital

e, Other (please specify…………………………..)

1. **What department do you work at?**

a, Anesthesiology and intensive care unit

b, Pain Clinic

c, Surgery

d, Traumatology

e, Orthopedics

1. **Which department or service deals with postoperative pain management in the hospital?**

(please mark all true answers)

a, Anesthesiology and intensive care unit

b, Acute Pain Service

c, Pain Clinic

d, Operating department

e, Other (please specify…………………………………………………………………………..)

**APS questions (to be filled in by an anesthesiologist!)**

1. **Do you have APS in your hospital?**

a, Yes.

b, No.

**(If your answer is no, please go to question 26.)**

1. **How long have you had APS in your hospital?**

a, Less than 5 years.

b, More than 5 years.

1. **How would you describe APS in your hospital?**

a, Struggling.

b, Flourishing.

c, Established.

1. **Who works in the APS team?**

a, Anesthesiologist

b, Anesthesiology assistant

c, Pharmacist

d, Surgeon

e, Neurologist

f, Ward nurse

g, Advanced Practice Registered Nurse

h, Pain nurse

i, Physiotherapist

j, Psychologist

k, Other (please specify………………………)

1. **How many anesthesiologists are involved in APS?**

a, 1

b, 2

c, 3 or more

d, Other (please specify…………………………..)

1. **How many anesthesiology assistants are involved in APS?**

a, 0

b, 1

c, 2

d, 3 or more

e, Other (please specify……………………………………………….)

1. **Do APS staff only treat postoperative pain?** (exclusively APS)

a, Yes, full time anesthesiologist.

b, No, part time anesthesiologist, performing the task alongside his/her other tasks.

c, Yes, fulltime anesthesiology assistant /nurse.

d, No, part time anesthesiology assistant /nurse performing the task alongside his/her other tasks.

e, Other (please specify…………………………………………)

1. **Do APS staff have the opportunity to consult a psychiatrist?**

a, Yes.

b, No.

1. **Do APS staff treat non-surgical patients as part of their work (e.g. obstetric, cancer, rheumatological pain)?**

a, Yes.

b, No.

1. **What is the average amount of APS work in the non-postoperative pain treatment (e.g. obstetric, cancer, rheumatological pain)?**

a, 0-20%

b, 20-40%

c, 40-60%

d, 60-80%

e, more than 80%

1. **How is APS funded?**

a, No funding.

b, Anesthesiology department receives special funding for it.

c, Anesthesiology department does not receive special funding for it, uses part of its own funds.

d, Surgical department receives funding for it.

e, Other (please specify……………………………………………………)

f, I have no information.

1. **Is there any written agreement between APS and the surgical department with regard to postoperative pain treatment?**

a, Yes.

b, No.

c, I have no information.

If yes, please specify the main content elements……………………………………………………………….

1. **Does the APS team treat the pain in all surgical patients or only in some of the patients?**

a, Yes, in all patients.

b, No, only in part of the patients.

If yes, what kind of patient groups? (please, mark all true answers)

- patients with EDA cannula and IV-PCA
- patients undergoing major abdominal or thoracic surgery
- patients with unsatisfactory pain therapy

1. **Does the APS team provide service out of hours?**

a, Yes.

b, No.

If no, please specify who treat inadequately relieved pain out of hours?

1. **Does the APS team provide service at weekends?**

a, Yes.

b, No.

If no, please specify, who treat inadequately relieved pain at weekends?

1. **Does the APS team use special observation sheet in your hospital?**

a, Yes.

b, No.

If yes, please specify the main content elements…………………………………………………

1. **Are there any data collected about the work of the APS team?**

a, Yes.

b, No.

c, I have no information.

If yes, in what way are the data collected?

- on paper
- on electronic programs
- both

1. **Are the generated data shared?**

a, Yes.

b, No.

If yes, who with?

- hospital management
- colleagues
- surgical ward professionals
- other hospitals
- other (please specify………………………………………………………………………………….)

1. **Are there any data collected with regard to patient visits?**

a, Yes.

b, No.

If yes, what kind of data are collected?

- patient’s gender, age
- type of surgery
- ASA classification of patients
- incision site
- type of anesthesia
- patient’s pain scores
- number and kind of side effects and adverse events
- quality of technique
- other (please specify………………………………………………………………………………..)

1. **In case there is no APS team in your hospital, do you feel the need to have one?**

a, Yes.

b, No.

1. **What are the greatest barriers to have an APS team? (please mark the most important 4 answers)**

a, Unavailable drugs, equipment.

b, Lack of financial resources.

c, Lack of initiative and motivation.

d, Lack of support from the management.

e, Lack of interest from colleagues.

f, Insufficient cooperation between professions.

g, Lack of human resources.

h, Other (please specify…………………………………………………………………………………………….)

1. **Would you actively take part in developing an APS in the future?**

a, Yes.

b, No.

c, No comment.

1. **In your opinion, is the anesthesiologist to take responsibility for the operation of APSs in hospitals?**

a, Yes.

b, No.

Please, justify any of your answers………………………………………………………………………………………
