# Supplementary material for: Acute Pain Service in Hungarian hospitals
Source: PLoS One. 2021 Sep 22;16(9):e0257585. doi: 10.1371/journal.pone.0257585 (PMC8457450; doi:10.1371/journal.pone.0257585)
Supplement: Attachment — Submitted filename: Response to Reviewers.docx [file pone.0257585.s005.docx]

R1. *Since this is a research article, I would expect more results based on statistical analyses (but it is not clear how the data was analyzed).*

This explorative survey was the first of this kind in Hungary, and, as APS is present only in a small number of Hungarian hospitals, it was not possible to carry out a comparative study with more profound statistical analysis. The purpose of this survey was to explore a starting situation, for which descriptive statistical methods are appropriate.

R2. *Abstract: I would avoid using the word “irksome” – burdensome?*

We have changed the text accordingly.

R3. *Introduction: It would be important to use the new/revised definition of pain (according to IAPS) in this paper. For a reference see:*
    [*https://www.iasp-pain.org/PublicationsNews/NewsDetail.aspx?ItemNumber=10475*](https://www.iasp-pain.org/PublicationsNews/NewsDetail.aspx?ItemNumber=10475) *doi: 10.1097/j.pain.0000000000001939*
R4. *Again, I would avoid using the word “irksome” – burdensome?*

We have changed the text.

R5. *Chronic pain should be defined as well (within parentheses)*

We have complemented the text.

R6. *I would like to read more about how acute (and chronic) pain can affect the central nervous system and brain (structures and functions) as well. 1-2 sentences with reference*

We have added some information with reference to the text as requested.

R7. *“they are also an important factor “– important factors (plural)*

We have corrected the text.

R8. *“Petti [7], the frequency of prolonged postoperative pain can be between 5% to 85%, depending on the type of the operation” –– Can you give some examples? Is there a specific type of operation which usually relates to prolonged postoperative pain?*

We have complemented the text.

R9. *“whose number worldwide was estimated to exceed 300 million per annum in 2012” – Is there any more recent data/reference available?*

We have looked for it again, but have not found more recent data. According to the database of EUROSTAT, the most recent data are from 2016, which are not complete due to the deficiency of data service. In the database of WHO we have not found any data regarding the number of operations.

R10. *“decreases the pain scores of patients” sounds a bit informal – The authors should rephrase this (e.g., decreases the pain intensity in patients)*

We have corrected the text.

R11. *The aim of our study: the authors should state here that the study was done using a survey (this should not be first mentioned in the Methods section)*

We have inserted the required sentence in the introductory section.

R12. *Methods: “We identified published questionnaires [1, 18, 19, 27-28], selected the relevant questions and translated and adapted these to the Hungarian context” – Were these questions translated back into English and compared to the original versions?*

We did not consider it necessary to translate the questions back into the original language as the language of filling out was Hungarian and the questions were adapted in a way to be interpretable in the Hungarian Health Care System. Furthermore, we did not want to carry out international comparison.

R13. *“The collected data were processed and analyzed by SPSS 24.0.” – citation needed here, this is not the proper way to report what software was used for the analysis.*

We have complemented the text.

R14. *The tables should be better formatted –They occupy to much space relative to the information they demonstrate. Words should not be separated in a Table unless it is absolutely necessary*

The tables have been changed, as requested.

R15. *The Methods section is missing a Data Analysis section. There is no information available about how the analysis was done, what methods were used exactly to analyze the questionnaires, and there is no indication if any or which assumptions were tested etc. It seems that the study is based mainly on descriptive statistics.*

As we have pointed out earlier, this explorative survey was the first of this kind in Hungary, and, as APS is present only in a small number of Hungarian hospitals, it was not possible to carry out a comparative study with more profound statistical analysis. The purpose of this survey was to explore a starting situation, for which descriptive statistical methods are appropriate.

R16. *Did the participants get any compensation for completing the survey? (if not this should be stated as well)*

The responders did not get any compensation. We have specified this in the manuscript, as requested.

R17. *Results: “Among the respondent hospitals without established APS (N=115), the overwhelming majority (82.6%, n=95) think that such multidisciplinary teams would be necessary, while less than one fifth (17.4%, n=20) think that they are not needed” – What do we know about these 2 groups in terms of position and speciality?*

We have complemented the text, as requested.

R18. *Pain scores should be defined throughout the paper (and under the Tables in the Notes section)*
R19. *Minimum and maximum values should be stated where applicable*

We have complemented the text accordingly.

R20. *Discussion: “From a cross-country comparative perspective, we can say that the wealthier European countries, with high level of public spending on health started the introduction of APS decades ago, and the technology has been gaining ground at a fast pace ever since.” – the authors need to use some references here. On what study is this statement based on?*

We have added the requested citations.

R21. *Limitation should be a separate section and then Conclusion should be the last section*

We have changed the text accordingly.

R22. *The quality of Figure 1 needs to be improved*

We have used PACE before submitting the manuscript to check the quality of our figure as suggested by the journal. Additionally, in the revised version we have adjusted the text to improve clarity. If any further correction is needed, please provide us more guidance on what to change.
