# Supplementary material for: Acute Pain Service in Hungarian hospitals
Source: PLoS One. 2021 Sep 22;16(9):e0257585. doi: 10.1371/journal.pone.0257585 (PMC8457450; doi:10.1371/journal.pone.0257585)
Supplement: Attachment — Submitted filename: Response to Reviewers.docx [file pone.0257585.s006.docx]

Our responses to the reviewers’ comments can be found below.

**Reviewer #1**

*However, for future submissions, the authors should highlight the changes they make in the revised text (it is very difficult for the reviewers to find all the newly added information in the text).*

*Also, when the authors write their responses, they should list where they made the corrections (on what page), moreover, they should show these changes even in the "Response to the reviewers' comments" document.*

*All in all, the revision process needs to be improved by the authors.*

We are sorry, that our responses were difficult to follow, we will work to improve our efficiency and clarity during future revisions. However, a marked-up copy of the manuscript that highlights changes made is a mandatory requirement during revision, and we prepared it accordingly, and attached to our submission, so we thought this was enough to highlight the changes. It would have been no problem for us to indicate the relevant page numbers, and include the actual changes in the “response document”. We were not aware that this was a requirement and happy to do so in the future, as we have done in this current document.

**Reviewer #2**

*The authors wrote an article about use of APS services using a survey among all the 72 Hungarian hospitals, which had surgical inpatient care.*

*Although I am not the peer reviewer that made the initial comments, I carefully read the article, the comments and the responses/changes. I think that the response of the review comments was adequate.*

*I think this is an interesting article and technically sound. As mentioned in limitations, only descriptive statistics were possible, because the Ministry of Human Capacities, managed the distribution and collection of questionnaires, and returned the completed questionnaires in one pile, without sorting them according to hospitals and only two hospitals had APS teams. However, I miss information about the characteristics of hospitals that did not respond? All private hospitals? And if information about respondents (of different wards in different hospitals) is given, please do not suggest that this is from hospitals (for example in page 13, line 252).*

Although, we did not detail it in the text, in Table 2 we added information about the Hungarian hospitals (included in the survey and responding). In total, 20 hospitals did not return filled questionnaires, 12 government hospitals, two university clinics, one specialized hospital (for professional athletes) and five private for-profit hospitals. This sentence was also added to the text, in lines 184-185.

Additionally, we have checked the text throughout regarding hospitals vs. departments/wards, and improved the clarity of the text as “Among the respondent departments from hospitals without established APS (N=115),” in line 252. We have not conquered this problem elsewhere in the text.

*Furthermore, the introduction is quite long, about 3,5 pages. I think this could be more concisely written.*

We have shortened the introduction as requested. However, we feel that the information included in the introduction is necessary to understand the topic for all readers who are interested but not specialized in pain management. Some of the additional content of the introduction was incorporated as a response to the request of reviewer 1.

The following sentences were deleted:

“Because pain causes suffering, it is a priority symptom among patients and its relief is an important determinant of patient satisfaction, so much so that professional organizations, such as the American Pain Society, labelled it the „fifth vital sign” in addition to body temperature, pulse rate, respiration rate and blood pressure [4-6].” (from lines 49-53)

“Prolonged pain can cause disability, whose costs are also high. In the USA, for instance, it was estimated to be 34 000 USD per patient per annum [1]”. (from lines 63-65)

“reducing length of stay, as well as to improve the quality of pain therapies through innovation” (from line 95)
